# Effects of supplemental 25-hydroxyvitamin D_3_ on growth performance, physiological responses, and gene expression of skeletal muscle growth of finishing beef cattle

**DOI:** 10.1093/jas/skaf090

**Published:** 2025-03-23

**Authors:** Tainá E Martins, Vinícius N Gouvêa, Alexandre Perdigão, Maria Betania Niehues, Cyntia L Martins, Danilo D Millen, Tiago S Acedo, Victor V Carvalho, Luis F M Tamassia, Mario D B Arrigoni

**Affiliations:** Department of Animal Production, School of Veterinary Medicine and Animal Science, São Paulo State University, Botucatu, São Paulo, SP, Brazil; Texas A&M AgriLife Research, Amarillo, TX 79106, USA; Department of Animal Science, Texas A&M University, College Station, TX 77845, USA; Department of Animal Production, School of Veterinary Medicine and Animal Science, São Paulo State University, Botucatu, São Paulo, SP, Brazil; Department of Animal Production, School of Veterinary Medicine and Animal Science, São Paulo State University, Botucatu, São Paulo, SP, Brazil; Department of Animal Production, School of Veterinary Medicine and Animal Science, São Paulo State University, Botucatu, São Paulo, SP, Brazil; Department of Animal Science, College of Technology and Agricultural Sciences, São Paulo State University, Dracena, São Paulo, SP, Brazil; DSM Nutritional Products, Innovation and Applied Science, São Paulo, SP, Brazil; DSM Nutritional Products, Innovation and Applied Science, São Paulo, SP, Brazil; DSM Nutritional Products, Innovation and Applied Science, São Paulo, SP, Brazil; Department of Animal Production, School of Veterinary Medicine and Animal Science, São Paulo State University, Botucatu, São Paulo, SP, Brazil

**Keywords:** calcium, gene expression, mammalian target of rapamycin, meat, skeletal muscle

## Abstract

The objective of this experiment was to evaluate the effects of 25-hydroxyvitamin D_3_ (25(OH)D_3_) supplementation on growth performance, carcass characteristics, meat quality, and the expression of genes related to anabolism of skeletal muscle in finishing beef cattle. One hundred and twenty Nellore bulls (initial body weight [**BW**] = 376 ± 20 kg) were blocked by initial BW, allocated to 24 pens (5 bulls/pen) and pens were assigned to one of three treatments during a 96 d feeding experiment: control: high-concentrate basal diet (11% roughage; NEg = 1.16 Mcal/kg dry matter) with no supplemental 25(OH)D_3_ (0 mg of 25(OH)D_3_; *n* = 8 pens); basal diet containing supplemental 25(OH)D_3_ to provide 1 mg/animal/d (1 mg of 25(OH)D_3_; *n* = 8 pens), 3) basal diet containing supplemental 25(OH)D_3_ to provide 3 mg/animal/d (3 mg of 25(OH)D_3_; *n* = 8 pens). The dietary supplementation of 25(OH)D_3_ did not affect final BW, dry matter intake, average daily gain, and feed efficiency (*P* ≥ 0.32). Dressing percentage increased quadratically (*P* = 0.03) and *Longissimus muscle* area tended to increase quadratically (*P* = 0.09) with increasing levels of 25(OH)D_3_ supplementation. A treatment × day interaction was observed for plasma concentration of 25(OH)D_3_ (*P* < 0.001). No difference in plasma 25(OH)D_3_ concentration between treatments was observed at the beginning of the experiment (*P* > 0.05), but on days 37 and 95, plasma 25(OH)D_3_ was greater (*P* ≤ 0.05) for bulls fed 3 mg, followed by 1 mg, and 0 mg of 25(OH)D_3._ No effects of dietary supplementation of 25(OH)D_3_ were observed on meat quality attributes (*P* ≥ 0.24), except for meat pH that linearly increased (*P* < 0.01). The percentage of fat in the carcasses decreased linearly (*P* = 0.03) with increasing levels of 25(OH)D_3_ supplementation, followed by a numerical increase (*P* = 0.11) in the percentage of muscle. The gene expression of insulin-like growth factor-2 (IGF2), mammalian target of rapamycin, and myostatin tended (*P* ≤ 0.10), and IGF1 increased linearly (*P* = 0.04) with increasing levels of 25(OH)D_3_. In summary, the inclusion of 25-hydroxyvitamin D_3_ in feedlot diets may go beyond regulating calcium metabolism and meat quality only. Dietary supplementation of 1 mg of 25(OH)D_3_ for finishing beef cattle increased carcass dressing percentage and *Longissimus muscle* area by the upregulation of genes associated with skeletal muscle growth insulin-like growth factor-1 and 2, mammalian target of rapamycin, and myostatin.

## Teaser

Supplementing 25-hydroxyvitamin D_3_ to finishing beef cattle increased carcass dressing percentage and the expression of genes regulating skeletal muscle growth, without affecting meat quality or growth performance.

## Introduction

Dietary vitamin D supplementation has been studied for many years in finishing feedlot cattle to increase meat quality, especially by decreasing shear force and improving tenderness (Swanek et al., 1999; Montgomery et al., 2000, 2004; Scanga et al., 2001; Foote et al., 2004; Carnagey et al., 2008; Duffy et al., 2017). The metabolite 25-hydroxyvitamin D_3_ (25(OH) D_3_) is the major circulating form of vitamin D, which is formed in the liver (Reinhardt and Hustmyer, 1987; NRC, 1996) and transported to the kidney where the active metabolite (1,25-dihydroxy cholecalciferol; 1,25(OH)_2_D_3_) is formed (NRC, 1996). The general function of vitamin D is to increase plasma calcium (Ca) and phosphorous (P) to a level that supports normal bone mineralization (Deluca, 1979). Still, according to DeLuca (2004), the parathyroid gland is the Ca-sensing organ, and through the actions of parathyroid hormone and the active form of vitamin D (1,25(OH)_2_D_3_), Ca and P are mobilized from the bone to the plasma. The improvement in meat tenderness due to vitamin D supplementation for finishing cattle is usually attributed to the activation of the calpain proteolytic system postmortem because of their Ca dependency (Koohmaraie et al., 1988; NASEM, 2016).

Recently, there has been a growing body of evidence pointing to the effect of vitamin D on muscle mass function using in vitro models. The Akt/mTOR pathway is a signaling pathway that regulates cell growth and proliferation (Ge and Chen, 2012) and is activated by anabolic factors such as insulin and leucine. According to Salles et al. (2013), 1,25(OH)_2_D_3_ can enhance the effect of leucine and insulin on skeletal muscle anabolism, resulting in significant protein synthesis in murine in vitro C2C12 myotubes. The mammalian target of rapamycin (**mTOR**) is a vital regulator of muscle growth (Laplante and Sabatini, 2012; Yoon, 2017). Vignale et al. (2015) observed that broilers fed supplemental 25(OH)D_3_ (5520 IU/kg feed) for 42 d had a 126% increase in circulating concentrations of 25(OH)D_3_, greater breast meat yield, and greater fractional rate of protein synthesis compared with broilers fed the control diet with normal cholecalciferol levels (2,760 IU/kg feed). Still, according to these same authors, broilers fed supplemental 25(OH)D_3_ had a greater expression of vitamin D receptor (**VDR**) protein, greater phosphorylated concentration of mTOR, and a greater in vitro expression of upregulated mTOR genes compared to the control group. To the best of our knowledge, no information is available on the effects of 25(OH)D_3_ on the expression of genes related to muscle mass growth in finishing beef cattle.

Based on the aforementioned research, we hypothesized that 25(OH)D_3_ supplementation would increase the growth performance of finishing Nellore bulls through an increase in the expression of anabolic genes associated with skeletal muscle synthesis. Our objective was to evaluate growth performance, carcass characteristics, meat quality and composition, and the expression of genes related to muscle anabolism and catabolism of finishing Nellore bulls supplemented with 25(OH)D_3_.

## Material and Methods

This study was conducted at the São Paulo State University (UNESP), School of Veterinary Medicine and Animal Science (FMVZ), in Botucatu, state of São Paulo, Brazil, between October and February. All the procedures involving animals were reviewed and approved by the Animal Care and Use Committee of the School of Veterinary Medicine and Animal Science of the UNESP (# 0067/2017).

### Animals, housing, and treatments

A total of one hundred and twenty Nellore bulls (*Bos indicus*; initial body weight [**BW**] = 376 ± 24.5 kg) were used in a randomized complete block design to evaluate the effects of 25(OH)D_3_ supplementation on growth performance, carcass characteristics, physiological responses, and meat quality of finishing bulls. Bulls were purchased from one single ranch and were raised in a continuous grazing system before the beginning of the study.

Upon arrival at the research feedlot (day −10) after approximately 6 h of road transportation, bulls were individually weighed (off-truck BW; KM3 Plus, Coimma, Dracena, São Paulo, Brazil), vaccinated against clostridiosis (5 mL s.c. injection, Poli-Star, MSD Saúde Animal, Campinas, São Paulo, Brazil) and dewormed with 2 mL per 50 kg BW of 10% albendazole (Abendator 10%, Fabiani Saúde Animal, São Paulo, Brazil), and submitted to a pretrial acclimatization period of 10 d to adapt bulls to the research facility. During this 10-d pretrial acclimatization period, bulls were fed a diet containing 77% Tifton 85 hay (*Cynodon dactylon*), 20% soybean meal; and 3% mineral supplement, and the diet was offered as total mixed ration at 2% (DM basis) of the average arrival off-truck BW.

At the beginning of the experiment on day 0, bulls were individually weighed after 16 h of feed and water withdrawal, identified with ear tags, blocked by shrunk BW, and allocated to 24 roofed pens (5 m × 5.35 m; *n* = 5 bulls per pen and 0.84 m of bunk space per bull). Pens within each BW block were then randomly allocated to one of three treatments: 1) control group: basal diet with no supplemental 25(OH)D_3_ (0 mg of 25(OH)D_3_; *n = *8 pens), 2) basal diet containing supplemental 25(OH)D_3_ to provide 1 mg/animal/d (1 mg of 25(OH)D_3_; *n* = 8 pens), 3) basal diet containing supplemental 25(OH)D_3_ to provide 3 mg/animal/d (3 mg of 25(OH)D_3_; *n* = 8 pens). The dose of 25(OH)D_3_ was based on Mudado et al. (2024) and Silva et al. (2022). The 25(OH)D_3_ (Rovimix Hy-D 1.25%) was provided by DSM Nutritional Products, Basel, Switzerland.

### Feeding management

The basal diet was formulated to meet the nutrient requirements of finishing bulls according to the Large Ruminant Nutrition System (Tedeschi and Fox, 2020; Table 1) for an average daily gain (**ADG**) of 1.45 kg/bull daily. Bulls were adapted to the high-concentrate diet during the initial 15 d of the experiment using two step-up diets which decreased the roughage level (sugarcane bagasse) from 24% (Adap1; 7 d) to 19% (Adap2; 8 d; Table 1). From days 16 to 43 bulls were fed a growing basal diet containing 14% roughage and 86% concentrate and from days 44 to 96 a finishing basal diet containing 11% roughage and 89% concentrate (Table 1). The 25(OH)D_3_ was incorporated or not (for the control group) into the mineral–vitamin supplement that was included at 4% of the dietary dry matter (**DM**) (Table 1) throughout the 96 d of the experiment. The mineral–vitamin supplements were produced at a commercial feed mill following all the manufacturing standards for quality and guaranteed concentration (e.g., guaranteed concentration and stability tests according to the local regulatory authorities; DSM Nutritional Products Brazil S.A., Mairinque, SP, Brazil), and only differed in the concentration of 25(OH)D_3_.

**Table 1.** Ingredient and analyzed chemical composition of diets (dry matter [DM] basis)

Item	Adap1	Adap 2	Growing	Finishing
Days in each diet	7	8	28	53
Ingredients, %				
Sugarcane bagasse	24.0	19.0	14.0	11.0
Ground corn	28.3	36.0	43.8	51.8
Pelleted citrus pulp	18.7	17.2	15.0	14.7
Soybean meal	12.3	9.80	4.50	4.50
Cottonseed cake	12.7	14.0	18.7	14.0
Mineral and vitamin supplement^1^	4.00	4.00	4.00	4.00
Analyzed chemical composition, %				
Dry matter	73.0	76.0	79.0	81.0
Crude protein	16.1	15.8	15.0	14.0
Neutral detergent fiber	34.9	31.5	29.0	25.3
Non-fiber carbohydrates^2^	39.0	43.0	46.0	50.5
Total digestible nutrients^2^	69.0	71.0	73.0	74.5
Net energy for gain, Mcal/Kg^2^	0.99	1.04	1.10	1.16

^1^Containing the dietary treatments: 0 mg = basal diet with no supplemental 25-hydroxyvitamin D_3_ (25(OH)D_3_; *n = *8 pens; 5 bulls/pen); 1 mg = basal diet containing supplemental 25(OH)D_3_ to provide 1 mg/animal/d (*n = *8 pens; 5 bulls/pen); 3 mg = basal diet containing supplemental 25(OH)D_3_ to provide 3 mg/animal/d (*n* = 8 pens; 5 bulls/pen). The 25(OH)D_3_ (Rovimix Hy-D 1.25%) was provided by DSM Nutritional Products, Basel, Switzerland.

^1^Custom mineral and vitamin supplement containing (DM basis): NNP = 700.00 g/kg; Ca (min) = 110.00 g/kg; Ca (max) = 130.00 g/kg; P (min) = 12.00 g/kg; K (min) = 25.00 g/kg; S (min) = 27.00 g; kg; Mg (min) = 5.00 g/kg; Na (min) = 42.00 g/kg; Co(min) = 6.00 mg/kg; Cu (min) = 400.00 mg/kg; Cr (min) = 5.00 mg/kg; I (min) = 20.00 mg/kg; Mn (min) = 800.00 mg/kg; Se (min) = 5.00 mg/kg; Zin (min) = 1,500.00 mg/kg; Vit A (min) = 125,000.00 IU/kg; VitD_3_ (min) = 12,500.00 IU/kg; Vit E (min) = 1,300.00 IU/kg; Biotin (min) = 67.00 mg/kg; α-amylase = 8,400.00 KNU/kg; D-Limonene = 855.00 mg/kg; *Saccharomyces cerevisiae* = 2 × 10^9^ CFU/kg; Fluoride (max) = 120.00 mg/kg; 25-hydroxyvitamin D_3_ = 0, 2.60 and 7.70 mg/kg, respectively for treatments 0, 1, and 3 mg of 25(OH)D_3_; Manufactured by DSM Nutritional Products, São Paulo, Brazil.

^2^Estimated using the Large Ruminant Nutrition System (Tedeschi and Fox, 2020).

Each treatment diet was mixed individually using a feed mixer equipped with electronic scale (Vertimix 20AC; Casale, São Carlos, SP, Brazil), weighed into 50 kg nylon bags using a fixed scale with 0.05 kg readability (2098, Toledo do Brazil, Sao Bernardo do Campo, SP, Brazil), and delivered manually to each pen twice daily at 0800 (60% of the total feed call) and 1500 hours (40% of the total feed call). Feed bunks were evaluated visually each day and managed for a maximum of 3% refusals daily. Refusals were removed daily before the morning feeding, weighed, and a subsample was dried at 105 °C for 24 h for DM determination and dry matter intake (**DMI**) calculation. Sample of the diets and feed ingredients were collected weekly for determination of DM (method 930.15; AOAC, 2006), crude protein (method 984.13; AOAC, 2006), and neutral detergent fiber using α-amylase and sodium sulfite (Van Soest et al., 1991); modified for use in an Ankom 200 fiber analyzer, Ankom Technology Corp.).

### Growth performance and carcass characteristics

Bulls were individually weighted at the beginning (day 0) and end (day 96) of the experiment after 16 h of feed and water withdrawal for ADG calculation. The DMI was calculated based on the amount of feed offered and the refusals and was expressed in kilograms and as a percentage of BW. Feed efficiency was calculated as the ratio of ADG to DMI (G:F).

The 12th rib fat thickness, biceps femoris fat thickness, *Longissimus muscle* (**LM**) area, and marbling were measured via ultrasound on days 0 and 96 following the method described by Perkins et al. (1992). The evaluations were performed according to the international standards of the Ultrasound Guidelines Councill (**UGC**), using an Aloka 500V (AL-500) real-time ultrasound equipped with a 17.2 cm / 3.50 MHz transducer with 72 crystals (Corometrics Medical Systems, Inc., Wallingford, CT) and vegetable oil as an acoustic coupling. The images were collected and analyzed using the BIA Pro Plus software (Designer Genes Technologies Brazil), by technicians certified by UGC. The 12th rib fat thickness daily gain, biceps femoris fat daily gain, and LM area daily gain were calculated as the difference between the two measurements divided by days on feed.

Upon shrunk BW assessment on day 96, bulls were transported for approximately 2 h to a commercial packing plant and slaughtered the following day. Hot carcass weight (**HCW**) was obtained after kidney, pelvic, and heart fat removal. The dressing percentage was calculated by dividing HCW by the final BW.

### Blood gas profile, plasma 25-hydroxyvitamin D_3,_ and Ca

Blood samples were collected from the same two bulls/pen, representing the average BW from each pen, from the jugular vein 3 ± 1 h after feeding on days −1, 37, and 95 using 2 mL syringes containing lithium heparin as an anticoagulant for blood gases, chemistry, and 25-hydroxyvitamin D_3_ analyses. Blood gases and chemistries (pH, bicarbonate [HCO_3_], total CO_2_ [TCO_2_], and glucose) were analyzed using a portable handheld blood analyzer (VetScan i-STAT 1; Abbott Laboratories, Abbott Park, IL) loaded with the i-STAT CG4+ cartridge (Abbott Laboratories).

Blood samples for the analysis of total Ca (tCa), ionized Ca (iCa), and 25-hydroxyvitamin D_3_, were collected into 10 mL collection tubes containing 158 USP units of sodium heparin as an anticoagulant for plasma separation (BD Vacutainer, Franklin lakes, NJ, EUA). After blood collection, samples were immediately placed on ice until centrifugation (approximately 30 min). The samples were centrifuged for 15 min at 2,500 × *g* (LS-3 Plus, Companhia Equipadora de Laboratórios Modernos, Barueri, Sao Paulo, Brazil), and plasma samples were transferred into multiple aliquots of 2.5 mL and stored at −20 °C for later analyses. Plasma tCa was determined using a commercially available endpoint colorimetric assay (Calcio Arsenazo III, Bioclin, Quimica Basica Ltda., Belo Horizonte, Minas Gerais, Brazil), and optical density was read at 650 nm using a spectrophotometer microplate reader with an inter CV lower than 5% (Eon, Biotek, Winooski, VT). The iCa was determined using the ion-selective technique with an automatic ion analyzer (MHlab-ise; MH Equipamentos e Materiais para Laboratório Ltda., Sao Paulo, Brazil), and 25(OH)D_3_ was analyzed using high-performance liquid chromatography coupled with mass spectrometry (LC-MS/MS) detection.

### Ca determination in muscle tissue

Samples of the right LM between the 11th and 12th rib were collected from the same two bulls/pen on day 95 as described by Schubach et al. (2019) and Cooke et al. (2023). Each LM sample was divided into two subsamples and immediately placed in two 2-mL microtubes, containing or not 1 mL of RNA stabilization solution (RNAlater, Ambion, Inc., Austin, TX), and were frozen at −80 °C until further processing for laboratory analyses.

For Ca determination, approximately 100 mg of sample, cryogenically ground, were transferred directly to Teflon vials in the microwave oven (DGT 100 plus, Provecto Analítica, Campinas, SP, Brazil) and later 2.5 mL of nitric acid (14 mol/L) plus 0.50 mL of hydrogen peroxide were added (30% w/w) for acid digestion. The acid extracts obtained were transferred to 25 mL volumetric flasks and the volumes were filled with ultrapure water. An atomic absorption spectrometer (model AA-6800) equipped with a background absorption corrector with a deuterium lamp and a self-reverse system was used to determine Ca. Absorbance readings were taken by the flame module (SpectrAA 220 FS Varian, Agilent Technologies, Santa Clara, CA), using acetylene as fuel gas and air as oxidizing gas, with a constant flow of 2 L/min.

### Gene expression of genes related to muscle anabolism and catabolism

LM samples stored in the microtube containing 1 mL of RNA stabilization solution were used to analyze genes related to muscle anabolism and catabolism. Quantitative evaluation of gene expression was performed by RT-qPCR reaction. The genes evaluated in this study were: antioxidant marker superoxide dismutase 1 (SOD1), genes related to protein synthesis (anabolism), insulin-like growth factor-1 and 2 (IGF1 and IGF2, respectively), and mammalian target of rapamycin (mTOR), and genes related to muscle catabolism, Forkanimal box protein O1 (FOXO1), muscle ring-finger 1 (MuRF1), Atrogin-1, and myostatin (MSTN; Table 2). Total RNA was extracted from frozen LM samples using the TRIzol Plus RNA Purification Kit (Invitrogen, Carlsbad, CA) as described by Cooke et al. (2023). Briefly, LM samples were homogenized with Polytron (Brinkmann, Westbury, NY) using 1 mL of TRIzol reagent for each 50 to 100 mg of tissue, and incubated for 5 minutes at 28 °C. The insoluble fraction was discarded, and 0.2 mL of chloroform was added to each 1 mL of TRIzol, and the solution was incubated for 3 minutes at 28 °C and then centrifuged at 1,200 rpm for 15 minutes at 4 °C, and the formed aqueous phase was separated. The RNA was precipitated with 0.5 mL of isopropyl alcohol for 10 minutes at 28 °C and centrifuged at 1,2000 rpm for 10 minutes at 4 °C. The RNA pellet formed was dried at 28 °C for 25 minutes, followed by resuspension in ultrapure water and finally stored at −80 °C. The NanoVueTM Plus spectrophotometer (GE Healthcare, Dallas, TX) was used to quantify the RNA, which also allowed an estimate of the extraction quality by measuring the absorbance of 260 nm (amount of RNA) and 280 nm (amount of proteins). The purity of the RNA was guaranteed by obtaining a ratio of 260/280 nm greater than 1.8 (Fleige and Pfaffl, 2006). The integrity (quality) of the total extracted RNA was assessed by 1% agarose gel electrophoresis. DNAse treatment of RNA-As per the DNAse I protocol instructions—Degree of amplification (Invitrogen Life Technologies, Carlsbad, CA), 1 µg of total RNA for the reverse transcription (**RT**) reaction was transferred to a sterile microfuge tube, which was added 1 µL of 10X DNAse I Reaction Buffer, 1 µL of DNAse I Amp Grade (1U/µL) and water-DEPC (treated with 0.01% diethylpyrocarbonate-DEPC-SIGMA) in sufficient quantity to complete 10 µL of solution. This solution remained at room temperature for 15 minutes and then 1 mL of EDTA (25 Mm) was added and incubated at 65 °C for 10 minutes for total inactivation of the DNAse I enzyme. The RT was performed using the high-capacity cDNA Archive kit (Life Technologies, Austin, TX). The expression of selected genes was detected by real-time PCR by the QuantStudioTM 7 Flex real-time PCR system (Applied Biosystems, Providence, RI). The cDNA of the genetic mRNAs was amplified using the Master Mix Power SYBRTM Green PCR kit (Applied Biosystems). Reactions for each gene were performed in duplicates. The thermocycling conditions of the qPCR reaction were standardized according to the equipment manufacturer’s instructions. For analysis and normalization of reactions, 2 reference genes were evaluated (PPARA and HPRT1) and HPRT1 was the one that showed greater stability being used for the normalization of gene expression data using the BestKeeper program (Pfaffl et al., 2004). The relative gene expression was calculated using the Pfalff method (Pfaffl, 2010). Results were analyzed and expressed as Delta delta C_T_ fold change (2^−ΔΔC^_T_) as described by Livak and Schmittgen (2001).

**Table 2.** Primer sets used for quantitative real-time reverse transcription polymerase chain reaction (qPCR) analysis in skeletal muscle of beef cattle supplemented or not with 25-hydroxyvitamin D

Target gene^1^	GenBank accession no.	Forward primer (5’ to 3’)	Reverse primer (5’ to 3’)
IGF1	NM_001077828.1	AGGATGTGATGGGCATCTTC	AAGCAATGGGAAAAATCAGC
IGF2	NM_174087.3	CAGGTTTGGGTCTTTGGTGT	TTGCAGGTAGGCTTGTCCTT
mTOR	XM_015466778.1	CCACGGACCAGTGAGGTAAT	TGGAGGACACGGATTAGGAC
MSTN	NM_001001525.3	TTGGGTTTTCCTTCCACTTG	GCTCCTTGGAAGACGATGAC
MuRF1	NM_001017951.1	CCTCAAACCTCTGGTTCAGC	TCACACAGATGGAGGAGGTG
Atrogin1	NM_001046155.1	ATCATCAGGGGAACCCTTCT	TTTCACTTTCACCCCAGGAC
SOD1	NM_174615.2	TCTCCAAACTGATGGACGTG	CTTCGAGGCAAAGGGAGATA
FOXO1	XM_015473787.1	CTGGGTGGACACAGTCAATG	CCCTGAGAACATGGAGAACC

^1^IGF-1: Insulin-like growth factor-1; IGF-2: Insulin-like growth factor-2; mTOR: mammalian target of rapamycin; MSTN: Myostatin; MuRF1: muscle ring-finger protein-1; Atrogin1: Atrogin-1; SOD1: Superoxide dismutase type 1; FOXO1: Forkanimal box protein O1.

### Meat quality and physical composition of the carcass

At 24 h postmortem, Longissimus thoracis muscle samples (approximately 25 cm long) from all bulls were collected from the left side of the carcasses from the proximal muscle section between the 12th and 13th ribs. Ribeye pH was measured using a digital portable pH meter (HI99163; Hanna Instruments Inc., Woonsocket, RI, USA) and samples were then cut into steaks approximately 2.54 cm thick, vacuum packaged in polyethylene bags, and aged for seven days for subsequent analysis of cooking losses, shear force, and color as described by Gouvêa et al. (2020) and Toseti et al. (2020).

Briefly, at the end of the 7-day aging period, samples were taken from the vacuum packages and left exposed for 30 min under refrigeration at a temperature of 4 to 6 °C before analyses.

#### Cooking losses:

 samples were identified, weighed, and roasted in a double-resistance industrial electric oven (Model F130 / L—Fornos Flecha de Ouro Ind. e Com. Ltda., São Paulo, Brazil) until an internal temperature of 71 °C. After cooling at room temperature at 25 °C, the samples were weighed again to determine the cooking loss as the percentage of weight lost during cooking (Honikel, 1998).

#### Warner-Bratzler shear force:

the tenderness was determined as recommended by the American Meat Science Association (AMSA, 2015). Samples were identified, weighed, roasted, and cooled as described above for cooking losses, and then wrapped in plastic film and placed in the refrigerator (4 to 6 °C) for 12 h. Eight cylinders, 1.27 cm in diameter, parallel to the longitudinal direction of the muscle fibers were removed to determine the Warner-Bratzler shear force through the TMS-PRO texture analyzer (Food Technology Corporation, Sterling, Virginia, USA). The shear force of each sample was considered as the average of the 8 repetitions (Wheeler et al., 1997).

#### Meat color:

samples were taken from the vacuum packages and left exposed for 30 min under refrigeration at 4 to 6 °C for objective color evaluation using the CIELAB system (CIE, 1986), carried out using a portable spectrophotometer, model CM2500d (Konica Minolta Brasil, Sãao Paulo, Brazil) with a standard D65 illuminant, 10° observation angle and 30 mm shutter aperture. The L* (luminosity), a* (red intensity), and b* (yellow intensity) values of each sample were obtained through an average of three measurements.

#### Physical composition of the carcass:

 the physical composition of the carcass was determined from 24 bulls/carcasses (1 bull/pen representing the average BW of each pen) according to the method proposed by Hankins and Howe (1946), using the 9th- to 11th-rib section (rib_9–11_) composition, on the left side of the carcass obtained 48 h after slaughter. The rib_9–11_ was dissected into bone, fat, and lean tissues (muscle). Each component was weighed and reported as a percentage of the total weight of the rib_9–11_.

### Statistical analysis

Data were analyzed as a randomized complete block design using the PROC MIXED of SAS 9.4 (SAS Inst., Cary, NC) and pen as the experimental unit. The model used to analyze growth performance, carcass characteristics, meat quality, muscle Ca concentration, and gene expression included the fixed effect of treatment and the random effect of block, pen, and steer (pen × treatment). Blood analytes were analyzed as repeated measures, and the model included the fixed effect of treatment, sampling day, and the resulting interaction between treatment × sampling day. Block, pen, and steer (pen × treatment) were included as random effects. The subject for the repeated statement was steer (pen × treatment), and the covariance structure utilized was autoregressive by providing the best fit according to the lowest Akaike information criterion. The Satterthwaite approximation method was used to determine the denominator degrees of freedom for tests of fixed effects. Orthogonal polynomial contrast statements (linear and quadratic) were constructed to determine the effects of treatments using Proc IML of SAS 9.4 since treatment levels were not equally spaced. If a treatment × sampling day interaction was detected, treatments were compared within each sampling day using Tukey–Kramer test. Data are reported as least square means. Significance was set at *P* ≤ 0.05 and tendencies were determined if *P* > 0.05 and ≤ 0.10.

## Results

Dietary supplementation of 25(OH)D_3_ did not affect final BW, DMI (kg and % of BW), ADG, or G:F (*P* ≥ 0.32; Table 3). Dressing percentage was quadratically increased (*P* = 0.03; Table 4), and LM area tended to increase quadratically (*P* = 0.09; Table 4) with increasing levels of 25(OH)D_3_ supplementation. No other effects of 25(OH)D_3_ supplementation were observed on carcass characteristics of finishing Nellore bulls (*P* ≥ 0.13; Table 4), despite the numerical increases in HCW, 12^th^ rib fat, and back-fat thickness for bulls supplemented with 1 mg of 25(OH)D_3_ (Table 4).

**Table 3.** Effects of supplemental 25-hydroxyvitamin D_3_ (25(OH)D_3_; mg/animal/d) on growth performance of finishing beef cattle

Item	25(OH)D_3_^1^			SEM^2^	*P*-value^3^	
	0 mg	1 mg	3 mg		Linear	Quadratic
Initial body weight, kg	376	376	376	8.47	-	-
Final body weight, kg	512	515	512	8.89	0.97	0.49
Dry matter intake, kg/animal	9.80	9.90	9.60	0.23	0.53	0.37
Dry matter intake, % of BW	2.21	2.24	2.16	0.05	0.40	0.32
Average daily gain, kg	1.42	1.46	1.41	0.05	0.96	0.49
Gain:Feed, kg/kg	0.144	0.145	0.147	0.003	0.35	0.89

^1^Treatments: 0 mg = basal diet with no supplemental 25(OH)D_3_ (*n = *8 pens; 5 bulls/pen); 1 mg = basal diet containing supplemental 25(OH)D_3_ to provide 1 mg/animal/d (*n = *8 pens; 5 bulls/pen); 3 mg = basal diet containing supplemental 25(OH)D_3_ to provide 3 mg/animal/d (*n* = 8 pens; 5 bulls/pen). The 25(OH)D_3_ (Rovimix Hy-D 1.25%) was provided by DSM Nutritional Products, Basel, Switzerland.

^2^Pooled standard error of the mean.

^3^Orthogonal polynomial contrasts: Linear = linear effect of 25(OH)D_3_ supplementation; Quadratic = quadratic effect of 25(OH)D_3_ supplementation.

**Table 4.** Effects of supplemental 25-hydroxyvitamin D_3_ (25(OH)D_3_; mg/animal/d) on carcass characteristics of finishing beef cattle

Item	25(OH)D_3_^1^			SEM^2^	*P*-value^3^	
	0 mg	1 mg	3 mg		Linear	Quadratic
Final body weight, kg	512	515	512	8.89	0.97	0.49
Hot carcass weight, kg	286	290	286	4.89	0.93	0.18
Dressing, %	55.9	56.4	55.9	0.20	0.94	0.03
Initial *Longissimus muscle* area, cm²	71.7	71.4	70.1	1.57	0.24	0.65
Final *Longissimus muscle* area, cm²	80.4	82.9	79.4	1.54	0.67	0.09
Initial marbling, %	2.87	2.76	2.77	0.098	0.49	0.62
Final marbling, %	3.10	3.00	2.90	0.10	0.21	0.89
Initial 12th rib fat, mm	3.02	3.07	3.01	0.112	0.94	0.68
Final 12th rib fat, mm	5.10	5.40	4.90	0.30	0.50	0.32
Initial back-fat thickness, mm	5.70	5.80	5.60	0.18	0.64	0.33
Final back-fat thickness, mm	8.10	8.60	8.00	0.29	0.70	0.13

^1^Treatments: 0 mg = basal diet with no supplemental 25(OH)D_3_ (*n = *8 pens; 5 bulls/pen); 1 mg = basal diet containing supplemental 25(OH)D_3_ to provide 1 mg/animal/d (*n = *8 pens; 5 bulls/pen); 3 mg = basal diet containing supplemental 25(OH)D_3_ to provide 3 mg/animal/d (*n* = 8 pens; 5 bulls/pen). The 25(OH)D_3_ (Rovimix Hy-D 1.25%) was provided by DSM Nutritional Products, Basel, Switzerland.

^2^Pooled standard error of the mean.

^3^Orthogonal polynomial contrasts: Linear = linear effect of 25(OH)D_3_ supplementation; Quadratic = quadratic efect of 25(OH)D_3_ supplementation.

No treatment × day interaction was observed for blood pH, bicarbonate, total CO_2_, and glucose (*P* ≥ 0.14: Table 5). Blood pH tended to decrease linearly (*P* = 0.09; Table 5) with increasing 25(OH)D_3_ supplementation. A sampling day effect was detected for blood pH and glucose levels (*P* < 0.01; Table 5), with blood pH increasing from 7.32 on day −1, to 7.38 on day 37, and 7.36 on day 95 (*P* < 0.05) and glucose decreasing from 103 mg/dL to 84.8 mg/dL, to 73.1 mg/dL from days −1, 37, and 95 respectively (*P* < 0.05). No effect of treatment, day, or treatment × day was observed for plasma concentration of bicarbonate and total CO_2_ concentration (*P* ≥ 0.18; Table 5).

**Table 5.** Effects of supplemental 25-hydroxyvitamin D_3_ (25(OH)D_3_; mg/animal/d) on blood chemistry and blood gas profile of finishing beef cattle

	25(OH)D_3_^1^			SEM^3^	Sampling day^2^			SEM^3^	*P*-value^4^		*P*-value^5^	
Item	Control	1 mg	3mg		−1	37	95		Linear	Quadratic	Day	25(OH)D_3 ×_ Day
pH	7.36	7.36	7.34	0.01	7.32^c^	7.38^a^	7.36^b^	0.008	0.09	0.45	<0.001	0.14
Bicarbonate, mmol/ L	24.6	24.7	24.3	0.29	24.7	24.6	24.3	0.25	0.29	0.51	0.67	0.24
Total CO_2,_ mmol/ L	25.8	25.7	25.6	0.33	26.0	25.7	25.3	0.28	0.33	0.90	0.18	0.24
Glucose, mg/ dL	86.0	84.4	90.4	3.29	103^a^	84.8^b^	73.1^c^	2.91	0.31	0.17	<0.001	0.95

^1^Treatments: 0 mg = basal diet with no supplemental 25(OH)D_3_ (*n = *8 pens; 2 bulls/pen); 1 mg = basal diet containing supplemental 25(OH)D_3_ to provide 1 mg/animal/d (*n = *8 pens; 2 bulls/pen); 3 mg = basal diet containing supplemental 25(OH)D_3_ to provide 3 mg/animal/d (*n* = 8 pens; 2 bulls/pen). The 25(OH)D_3_ (Rovimix Hy-D 1.25%) was provided by DSM Nutritional Products, Basel, Switzerland.

^2^Blood samples were collected from one bull/pen from the jugular vein 3 ± 1 h after feeding on days −1, 37, and 95 of the project.

^3^Pooled standard error of the mean.

^4^Orthogonal polynomial contrasts: Linear = linear effect of 25(OH)D_3_ supplementation; Quadratic = quadratic effect of 25(OH)D_3_ supplementation.

^5^Day = Effect of sampling day; 25(OH)D_3_ × Day = interaction between supplemental 25(OH)D_3_ and sampling day.

^abc^Main effect of sampling day—means within the same line without common superscript differ using *Tukey* test (*P* < 0.05).

**Figure 1.**
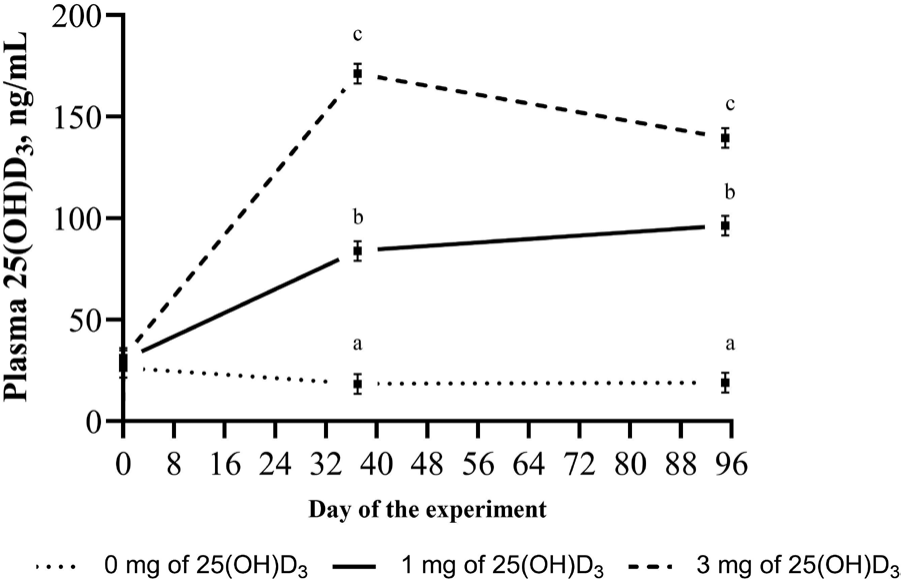
Effects of supplemental 25-hydroxyvitamin D_3_ (25(OH)D_3_; mg/animal/d) during a 96-d finishing feedlot on plasma concentration of 25(OH)D_3_ (ng/mL). Blood samples were collected on days −1, 37, and 95 of the experiment from the same 2 bulls/pen representing the average BW of each pen. A treatment × day interaction was detected (*P* < 0.001; SEM = 4.88). ^abc^Means within the same sampling day without common superscript differ using the *Tukey* test (*P* < 0.05).

A treatment × day interaction was observed for plasma concentration of 25(OH)D_3_ (*P* < 0.001; Table 6). No difference in plasma 25(OH)D_3_ concentration between treatments was observed at the beginning of the experiment (average of 29.3 ng/mL, *P* > 0.05; Fig. 1), but on days 37 and 95 of the experiment, plasma 25(OH)D_3_ was greater (*P* < 0.05) for bulls fed 3 mg, followed by 1 mg, and 0 mg of 25(OH)D_3_ (*P* < 0.05; Fig. 1)_._

**Table 6.** Effects of supplemental 25-hydroxyvitamin D_3_ (25(OH)D_3_; mg/animal/d) on plasma concentration of 25(OH)D_3_, total Ca (tCa) and ionized Ca (iCa), and muscle Ca concentration of finishing beef cattle

Item	25(OH)D_3_^1^			SEM^3^	Sampling day^2^			SEM^3^	*P*-value^4^		*P*-value^5^	
	0 mg	1 mg	3 mg		−1	37	95		Linear	Quadratic	Day	25(OH)D_3_ × Day
25(OH)D_3_, ng/mL	21.3	70.1	114	3.98	29.3	91.4	85.0	3.31	<0.001	0.57	<0.001	<0.001
Total Ca, μg/mL	190	197	204	5.17	174^c^	216^a^	202^b^	4.92	0.04	0.66	<0.001	0.08
Ionized Ca, µg/mL	29.4	30.4	31.2	1.03	33.2^a^	30.7^b^	26.9^c^	1.03	0.13	0.68	<0.001	0.12
Muscle Ca, µg/g	46.3	102	73.3	11.6	-	-	-		0.20	0.002	-	-

Treatments: 0 mg = basal diet with no supplemental 25(OH)D_3_ (*n = *8 pens; 2 bulls/pen); 1 mg = basal diet containing supplemental 25(OH)D_3_ to provide 1 mg/animal/d (*n = *8 pens; 2 bulls/pen); 3 mg = basal diet containing supplemental 25(OH)D_3_ to provide 3 mg/animal/d (*n* = 8 pens; 2 bulls/pen). The 25(OH)D_3_ (Rovimix Hy-D 1.25%) was provided by DSM Nutritional Products, Basel, Switzerland.

^2^Blood samples were collected from one bull/pen from the jugular vein 3 ± 1 h after feeding on days −1, 37, and 95 of the project.

^3^Pooled standard error of the mean.

^4^Orthogonal polynomial contrasts: Linear = linear effect of 25(OH)D_3_ supplementation; Quadratic = quadratic effect of 25(OH)D_3_ supplementation.

^5^Day = blood samples were collected from the jugular vein 3 ± 1 h after feeding on days −1, 37, and 95 of the project. 25(OH)D_3_ × Day = interaction between supplemental 25(OH)D_3_ and sampling days.

^abc^Main effect of sampling day—means within the same line without common superscript differ using *Tukey* test (*P* < 0.05).

Plasma concentration of total Ca (*P* = 0.04) increased linearly, and Ca concentration in the muscle increased quadratically (*P* = 0.002) with increasing levels of dietary 25(OH)D_3_ supplementation (Table 6). The plasma concentration of ionized Ca was not affected by dietary supplementation of 25(OH)D_3_ (*P* ≥ 0.13; Table 6). A sampling day effect was detected for plasma total and ionized Ca (*P* < 0.001; Table 6). Total Ca was greater on days 37 and 95 compared to day −1 (*P* < 0.05), and ionized Ca was greater on day -1, followed by days 37 and 95 (*P* < 0.05; Table 6).

No effects of dietary supplementation of 25(OH)D_3_ were observed on meat quality attributes (*P* ≥ 0.24), except for meat pH that linearly increased (*P* < 0.01) with increasing levels of 25(OH)D_3_ (Table 7).

**Table 7.** Effects of supplemental 25-hydroxyvitamin D_3_ (25(OH)D_3_; mg/animal/d) on meat quality of finishing beef cattle

Item	25(OH)D_3_^1^			SEM^2^	*P*-value^3^	
	0 mg	1 mg	3 mg		Linear	Quadratic
pH	5.71	5.80	5.97	0.06	<0.01	0.56
Shear force, kg/cm^2^	4.15	4.19	3.95	0.21	0.50	0.60
Cooking loss, %	19.5	19.7	20.3	0.79	0.50	0.84
Colour^4^						
L*	40.9	39.5	40.5	0.82	0.75	0.24
a*	13.4	13.0	13.2	0.54	0.72	0.68
b*	9.12	8.46	8.85	0.38	0.63	0.28

^1^Treatments: 0 mg = basal diet with no supplemental 25(OH)D_3_ (*n = *8 pens; 5 bulls/pen); 1 mg = basal diet containing supplemental 25(OH)D_3_ to provide 1 mg/animal/d (*n = *8 pens; 5 bulls/pen); 3 mg = basal diet containing supplemental 25(OH)D_3_ to provide 3 mg/animal/d (*n* = 8 pens; 5 bulls/pen). The 25(OH)D_3_ (Rovimix Hy-D 1.25%) was provided by DSM Nutritional Products, Basel, Switzerland.

^2^Pooled standard error of the mean.

^3^Orthogonal polynomial contrasts: Linear = linear effect of 25(OH)D_3_ supplementation; Quadratic = quadratic effect of 25(OH)D_3_ supplementation.

^4^ L* = brightness (0 = black, 100 = white), a* = redness (positive values = red, negative values = green), b* = yellowness (positive values = yellow, negative values = blue).

The percentage of fat in the carcasses decreased linearly (*P* = 0.03) with increasing levels of 25(OH)D_3_ supplementation, followed by a numerical increase (*P* = 0.11) in the percentage of muscle, with no effect of 25(OH)D_3_ supplementation on the percentage of bone in the carcasses (*P* ≥ 0.59; Table 8).

**Table 8.** Effects of supplemental 25-hydroxyvitamin D_3_ (25(OH)D_3_; mg/animal/d) on carcass physical composition estimated on the 9th- to 11th-rib section

Item, %	25(OH)D_3_^1^			SEM^2^	*P*-value^3^	
	0 mg	1 mg	3 mg		Linear	Quadratic
Bone	17.1	17.3	17.7	0.76	0.59	0.87
Fat	31.4	28.7	28.8	0.91	0.03	0.15
Muscle	51.3	53.8	53.3	0.86	0.11	0.16

^1^Treatments: 0 mg = basal diet with no supplemental 25(OH)D_3_ (*n = *8 pens; 1 bull/pen); 1 mg = basal diet containing supplemental 25(OH)D_3_ to provide 1 mg/animal/d (*n = *8 pens; 1 bull/pen); 3 mg = basal diet containing supplemental 25(OH)D_3_ to provide 3 mg/animal/d (*n* = 8 pens; 1 bull/pen). The 25(OH)D_3_ (Rovimix Hy-D 1.25%) was provided by DSM Nutritional Products, Basel, Switzerland.

^2^Pooled standard error of the mean.

^3^Orthogonal contrasts: Linear = linear effect of 25(OH)D_3_ supplementation; Quadratic = quadratic effect of 25(OH)D_3_ supplementation.

**Figure 2.**
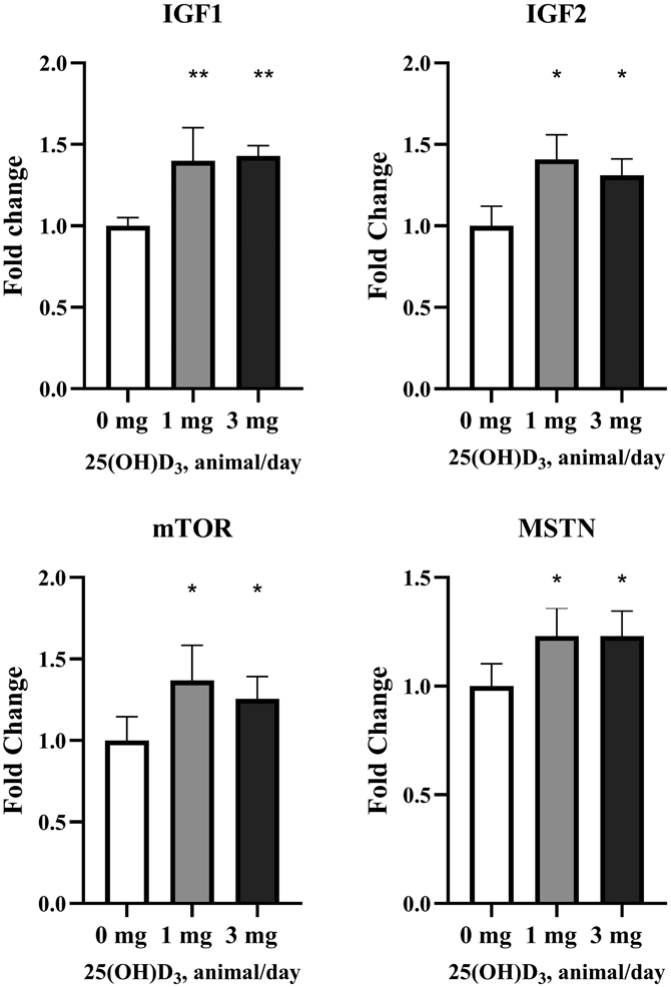
Fold change of genes regulating skeletal muscle growth in Nellore bulls fed diets containing increasing levels of supplemental 25(OH)D_3_ during a 96-d finishing feedlot. Samples of the right *Longissimus muscle* between the 11th and 12th rib were collected on day 95. 0 mg = basal diet with no supplemental 25(OH)D_3_ (*n* = 8 pens; 5 bulls/pen); 1 mg = basal diet containing supplemental 25(OH)D_3_ to provide 1 mg/animal/d (*n* = 8 pens; 5 bulls/pen); 3 mg = basal diet containing supplemental 25(OH)D_3_ to provide 3 mg/animal/d (*n* = 8 pens; 5 bulls/pen). IGF1, insulin-like growth factor-1; IGF2, insulin-like growth factor-2; mTOR: mammalian target of rapamycin; MSTN, Myostatin. **Indicates linear effect of 25(OH)D_3_ (*P* ≤ 0.04); *Indicates linear effect of 25(OH)D_3_ (0.05 < *P* ≤ 0.09).

**Figure 3.**
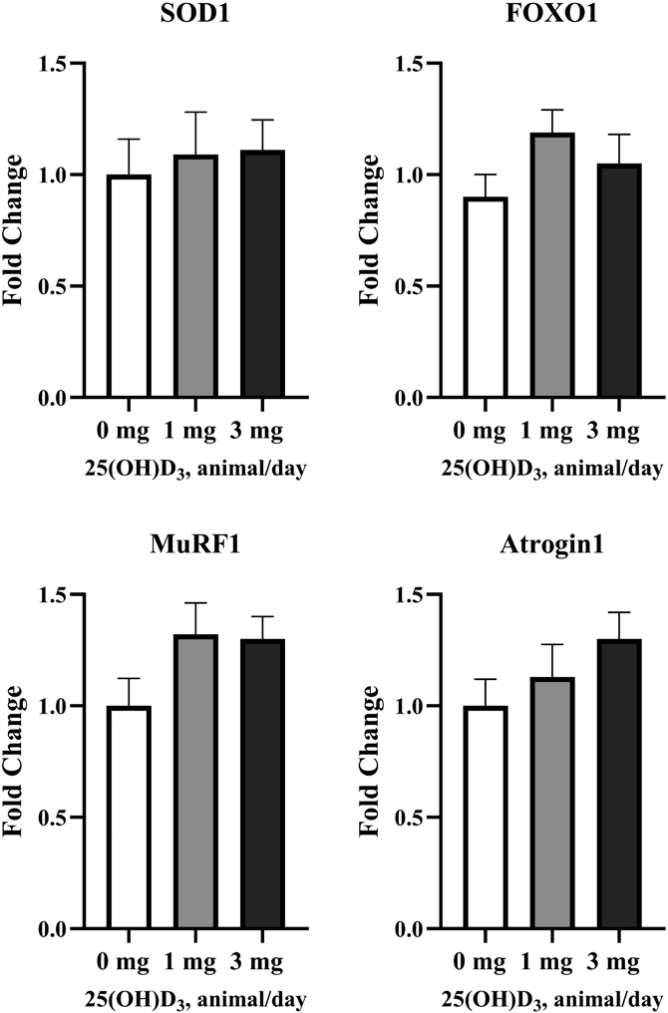
Fold change of genes regulating skeletal muscle growth in Nellore bulls fed diets containing increasing levels of supplemental 25(OH)D_3_ during a 96-d finishing feedlot. Samples of the right *Longissimus muscle* between the 11th and 12th rib were collected on day 95. 0 mg = basal diet with no supplemental 25(OH)D_3_ (*n* = 8 pens; 2 bulls/pen); 1 mg = basal diet containing supplemental 25(OH)D_3_ to provide 1 mg/animal/d (*n* = 8 pens; 2 bulls/pen); 3 mg = basal diet containing supplemental 25(OH)D_3_ to provide 3 mg/animal/d (*n* = 8 pens; 2 bulls/pen). MuRF1, muscle ring-finger protein-1; Atrogin1, Atrogin-1; SOD1, superoxide dismutase type 1; FOXO1, Forkanimal box protein O1. No effects of dietary supplementation of 25(OH)D_3_ were detected (*P* ≥ 0.05).

The gene expression of IGF2, mTOR, and MSTN tended to (*P* ≤ 0.07; Fig. 2) and IGF1 increased linearly (*P* = 0.04; Fig. 2) with increasing dietary levels of 25(OH)D_3_. No other effects on gene expression were detected (*P* ≥ 0.05; Fig. 3).

## Discussion

Vitamin D is well known to regulate Ca and P absorption, supporting normal mineralization of bone (DeLuca, 2004). Dietary supplementation of vitamin D for feedlot cattle has long been associated with meat quality, as a nutritional tool to decrease shear force and improve tenderness by a Ca-dependent proteolytic system postmortem (Swanek et al., 1999; Montgomery et al., 2000, 2004; Scanga et al., 2001; Foote et al., 2004; Duffy et al., 2017). Swanek et al. (1999) reported that feeding 125 to 185 mg/head daily of vitamin D (cholecalciferol) to finishing steers for 7 to 10 d before slaughter increased blood Ca and decreased shear force and sensory tenderness ratings. Duffy et al. (2017) also observed a decrease in shear force when steers were fed 0.9 mg/head daily for 30 d before slaughter. Previous research on dietary supplementation of vitamin D for feedlot cattle didn’t report an increase in growth performance with increasing levels of vitamin D supplementation (Swanek et al., 1999; Montgomery et al., 2000; Scanga et al., 2001; Duffy et al., 2017). These previous studies evaluated the dietary supplementation of cholecalciferol during the last few days of the feeding period before shipping cattle to slaughter (between 30 and 2 d before slaughter), and an increase in DMI or ADG was not expected due to the short time of vitamin D supplementation (Swanek et al., 1999). Karges et al. (2001) reported an 11.7% decrease in DMI on day 4 when steers were fed 150 mg of vitamin D_3_ during 4 d before slaughter. In the present experiment, the metabolite 25(OH)D_3_ was supplemented for 96 d and didn’t affect DMI or ADG. In broiler chickens, the metabolite 25(OH)D_3_ increased BW gain and feed efficiency compared to the same level of dietary supplementation of cholecalciferol (Yarger et al., 1995; Mudado et al., 2024), which supports the idea that the metabolite 25(OH)D_3_ is more efficient than cholecalciferol to increase BW gain in monogastric, but data comparing cholecalciferol and 25(OH)D_3_ on performance of beef cattle is scare. Recently, Mudado et al. (2024) reported an increase in the growth performance of grazing beef cattle supplemented with a protein-energy supplement containing 1 mg/animal/d of 25(OH)D_3_ compared to no 25(OH)D_3_ supplementation.

The increase in blood concentration of 25(OH)D_3_ and Ca due to increasing levels of dietary 25(OH)D_3_ in the current experiment agrees with previous research on beef (Foote et al., 2004; Duffy et al., 2017) and dairy (Silva et al., 2022) cattle. Similar to previous findings, our results confirm that 25(OH)D₃ is tightly regulated by the body. Blood Ca concentration is maintained at relatively constant levels by homeostatic mechanisms, even when the animal is fed a non-Ca diet (DeLuca, 2004), and it is usually not a good indicator of Ca status (NASEM, 2016). Blood 25(OH)D_3_ concentration is considered the best measure of vitamin D status, according to DeLuca (2004). Foote et al. (2004) administered one single bolus of 125 mg of 25(OH)D_3_ to steers 4 d before slaughter and observed a 27% increase in plasma Ca concentration at harvesting. The plasma concentration of 25(OH)D_3_ in this last study increased from 62.66 ng/mL in the control group (no supplemental vitamin D) to 269 ng/mL in steers that received the single bolus of 125 mg of 25(OH)D_3_. The increase in blood concentration of 25(OH)D_3_ due to vitamin D (cholecalciferol) or 25(OH)D_3_ dietary supplementation seems to be very consistent across all the published data. Changes in muscle Ca concentration due to 25(OH)D_3_ supplementation are variable. Contrary to our findings, Foote et al. (2004) didn’t observe differences in Ca concentration in LM by dietary supplementation of cholecalciferol or 1,25(OH)_2_D_3_, but a difference in shear-force due to dietary 25(OH)D_3_ supplementation was observed by these last authors. Montgomery et al. (2004) observed that *Bos indicus* cattle had greater 1,25-dihydroxyvitamin D_3_ (1,25-(OH)_2_D_3_) in tissues and plasma concentrations than *Bos taurus* cattle, which indicates that breed type can affect the need for vitamin D supplementation.

The increase in blood concentration of 25(OH)D_3_ and Ca, and Ca in the muscle in the current study due to the increasing levels of dietary 25(OH)_3_ supplementation agrees with the theory of myofibrillar proteolysis to increase tenderization as a result of Ca-dependent proteases (calpains; Edmunds et al., 1991; Koohmaraie, 1992), although shear force was not affected by 25(OH)D_3_ supplementation in the current experiment. Increasing levels of 25(OH)D_3_ supplementation increased meat pH in the current experiment, with pH values ranging from 5.71 to 5.97. According to Purchas et al. (1999) and Zhao et al. (2022), when meat pH is between 5.8 and 6.2, higher shear force values and less calpain activity are observed compared to normal (5.4 to 5.8) or high pH (>6.2). This could explain why, even with an increase in blood Ca concentration, differences in shear force were not observed in the current study.

The quadratic increase in carcass dressing percentage and LM area daily gain along with the linear decrease in the percentage of fat and numerical linear increase in the percentage of muscle observed in the carcasses of bulls supplemented with 25(OH)D_3_ is supported by the increase of genes associated with skeletal muscle growth IGF-1, IGF-2, mTOR, and MSTN observed herein. These findings are in agreement with recent data from Salles et al. (2013) and Vignale et al., (2015). The 1,25(OH)_2_D_3_ can enhance the effect of leucine and insulin on skeletal muscle anabolism, resulting in significant protein synthesis in murine in vitro C2C12 myotubes (Salles et al., 2013), The mammalian target of rapamycin (mTOR) is a vital regulator of muscle growth (Laplante and Sabatini, 2012; Yoon, 2017). A single VDR can mediate all the functions of vitamin D (DeLuca, 2004). According to Bischoff et al. (2001), VDRs were detected in skeletal muscle tissue. Salles et al. (2013) observed that 1,25(OH)_2_D_3_ can increase the expression of VDR in skeletal muscle cells in murine C2C12 myotubes. Still, according to these last authors, the gene expression of insulin receptors increased under the effect of 1,25(OH)_2_D_3_, along with its concentration and activation state, which could account for the anabolic effect of vitamin D on muscle cells. The critical role of calcium in regulating activity-dependent muscle gene expression has been well established in previous studies (Berchtold et al., 2000; Chin, 2005), which aligns with the increase in muscle calcium observed in the current study. Vignale et al. (2015) observed an increase in breast meat yield in broiler chickens supplemented with 25(OH)D_3_, but similarly to the current experiment, no differences in ADG were detected by Vignale et al. (2015). Still, according to these last authors, broilers fed supplemental 25(OH)D_3_ had a greater expression of vitamin D receptor (VDR) protein, greater phosphorylated concentration of mTOR, and a greater in vitro expression of upregulated mTOR genes compared to the control group. According to Oku et al. (2016), vitamin D restriction can decrease the mRNA expression of myogenic differentiation factor-1 (MyoD), a critical factor in muscle growth and differentiation (Rudnicki et al., 1993). Montenegro et al. (2019) observed a positive effect of vitamin D on in vitro myogenesis mediated by insulin.

Vitamin D deficiency is usually associated with increased obesity in humans (Vimaleswaran et al., 2013). The decrease in fat and increase in muscle observed in the carcass composition in the current experiment is somewhat in agreement with previous research on hypovitaminosis D and the incidence of obesity in humans. According to Wamberg et al., (2013), visceral and hepatic fat accumulation is inversely associated with plasma 25(OH)D_3_, and 1,25(OH)_2_D_3_ can inhibit adipogenesis in vitro. Because the requirements of vitamin D in ruminants were exceeded in the present study, and steers were relatively lean, with carcasses averaging 30% fat, when compared to a yield grade 5 carcass, which could show more than 35% fat as reported by Kelly et al. (1968), the main driver for the numerical increase in muscle observed in the present study is probably a direct effect of 25(OH)D_3_ on genes associated with skeletal muscle growth.

## Conclusions

In summary, the inclusion of 25-hydroxyvitamin D_3_ in feedlot diets may go beyond regulating Ca metabolism and meat quality only. Dietary supplementation of 1 mg of 25(OH)D_3_ for finishing beef cattle increased carcass dressing percentage and *Longissimus muscle* area by the upregulation of genes associated with skeletal muscle growth insulin-like growth factor-1 and 2, mammalian target of rapamycin, and myostatin.

## Abbreviations

1,25(OH)_2_D_3_: 1,25-dihydroxy cholecalciferol
25(OH)D_3_: 25-hydroxyvitamin D_3_
ADAP: adaptation ration
ADF: acid detergent fiber
ADG: average daily gain
BW: body weight
Ca: calcium
CO_2_: carbon dioxide
CP: crude protein
DE: digestible energy
DM: dry matter
DMI: dry matter intake
EE: ether extract
EG: energy of gain
FMVZ: School of Veterinary Medicine and Animal Science
FOXO1: Forkhead box protein 01
G:F: feed efficiency
GE: gross energy
HCO_3_: bicarbonate
HCW: hot carcass weight
iCa: ionized calcium
IGF1: insulin-like growth factor-1
IGF2: insulin-like growth factor-2
LC-MS/MS: liquid chromatography coupled with mass spectrometry
LM: Longissimus muscle
MDGS: modified distillers grains plus solubles
ME: metabolized energy
mRNA: messenger ribonucleic acid
mTOR: mammalian target of rapamycin
MuRF1: muscle RING-finger 1
MyoD: myogenic differentiation factor-1
N: nitrogen
NDF: neutral detergent fiber
NE: net energy
NEg: net energy of gain
NEm: net energy of maintenance
OM: organic matter
P: phosphorous
RNA: ribonucleic acid
SOD1: antioxidant marker superoxide dismutase 1
tCA: total calcium
TCO_2_: total CO_2_
UGC: Ultrasound Guidelines Councill
UNESP: Sao Paulo State University
VDR: vitamin D receptor
